# Occurrence of Recurrent Aphthous Stomatitis (RAS) as a Rare Oral Manifestation in a Patient with Gilbert's Syndrome

**DOI:** 10.1155/2021/6648729

**Published:** 2021-04-16

**Authors:** Artak Heboyan, Anna Avetisyan, Hans Erling Skallevold, Dinesh Rokaya, Vinay Marla, Anna Vardanyan

**Affiliations:** ^1^Department of Prosthodontics, Faculty of Stomatology, Yerevan State Medical University, Str. Koryun 2, Yerevan 0025, Armenia; ^2^Department of Therapeutic Dentistry, Faculty of Stomatology, Yerevan State Medical University, Str. Koryun 2, Yerevan 0025, Armenia; ^3^Christiania Tannlegesenter, Grønland 4, 0188 Oslo, Norway; ^4^Department of Clinical Dentistry, Walailak University International College of Dentistry, Bangkok 10400, Thailand; ^5^Department of Oral Pathology, Penang International Dental College, Penang 12000, Malaysia

## Abstract

Recurrent aphthous stomatitis is an ulcerative disease of the oral cavity and can occur in isolation or as a manifestation of many systemic diseases. It is a quite common entity and may hence often be overlooked as an isolated lesion. Gilbert's syndrome is a genetic disorder where a deficiency of an enzyme associated with the conjugation of bilirubin results in unconjugated hyperbilirubinemia. The disease is generally asymptomatic and is aggravated by certain trigger factors. No associated oral manifestations are known. In this case report, we discuss the concomitant presence of recurrent aphthous stomatitis in a patient of Gilbert's syndrome. The presence of such recurrent stomatitis may represent as an oral manifestation of Gilbert's syndrome. Early identification of these entities may improve the overall quality of life of the patient.

## 1. Introduction

Recurrent aphthous stomatitis (RAS) is a chronic inflammatory ulcerative disease of the oral cavity, manifested with the formation of one or several aphthae in the oral mucosa [1]. The recurrence of the ulcers has no clear pattern, yet they are characterized by long-term course, lasting for several years. The disease affects 5% to 60% of the world population, affecting mostly females [2]. RAS manifests in several systemic diseases [3] and may be the initial symptoms in some. The presence of aphthae may contribute in the diagnosis of an underlying disease [1].

Recurrent aphthous stomatitis is further classified, based on lesion size and number, into three types, namely, recurrent aphthous stomatitis minor, recurrent aphthous stomatitis major, and herpetiform ulcers [4]. Minor RAS is the most common type and manifests as numerous ulcers measuring 8 to 10 mm in diameter which heal within 2 weeks without scarring [5]. Major RAS, on the other hand, are lesions larger than 1 cm in diameter, multiple, and require a longer duration to heal. These ulcers generally scar upon healing [1, 5]. Herpetiform ulcers presents as multiple crops (up to 100) of painful, small (2-3 mm in diameter) ulcers but later coalesce to form larger ones. This variant is more common in older women and predominantly affects the tongue and floor of the mouth [6].

Diagnosis of recurrent aphthous stomatitis is mainly made on the basis of history and clinical presentation. Histopathology may be prescribed to rule out any other chronic ulcerative pathology [7]. The presence of systemic disease is confirmed based on the findings related to that pathology. The common systemic diseases associated with recurrent aphthous stomatitis include Behcet's syndrome, Magic syndrome, Sweet's syndrome, cyclic neutropenia, and acquired immunodeficiency syndrome (AIDS) [8]. The association of RAS with Gilbert's syndrome has, to the authors' knowledge, not been reported so far in the literature.

Gilbert's syndrome is a genetic disorder which is caused due to the deficiency of the enzyme uridine diphosphoglucuronate-glucuronosyltransferase 1A1 (UGT1A1) which is involved in the process of conjugation of bilirubin, thereby resulting in unconjugated hyperbilirubinemia [9]. Gilbert's syndrome is generally asymptomatic and is aggravated in association with certain triggering factors [10]. Oral manifestations of Gilbert's syndrome have not been well documented. A metallic bitter taste sensation has been reported by some patients [11]. This case report presents an unusual case of RAS, highlighting its concurrence with Gilbert's syndrome. The objective of this case report is to highlight a possible association of RAS and Gilbert's syndrome which would aid in early identification of this condition.

## 2. Case Presentation

A 20-year-old male presented with general weakness, pain in the right hypochondrium, lip pain, difficulty of food intake, and speech. A thorough general physical, extraoral, and intraoral examination was performed. The patient was of normosthenic build. On external body examination, visible integuments/skin were yellowish. No enlarged lymph nodes were detected. Auscultation of the heart and lungs revealed, respectively, a regular heart rate with clear tones and bilateral vesicular breathing sound. Blood pressure measured 110/70 mm Hg, and pulse was 77 beats per minute. On palpation and percussion, the abdomen was painless and slightly inflated, the Pasternatsky sign was negative bilaterally, and urination was voluntary.

**Figure 1:**
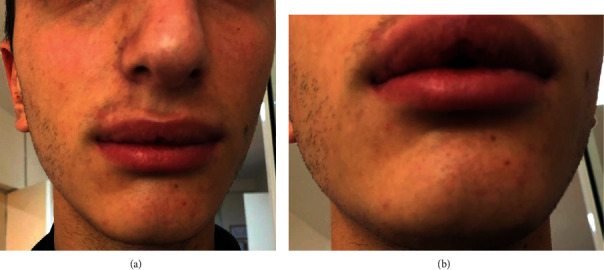
(a, b) Significant swelling of the upper and lower lips.

**Figure 2:**
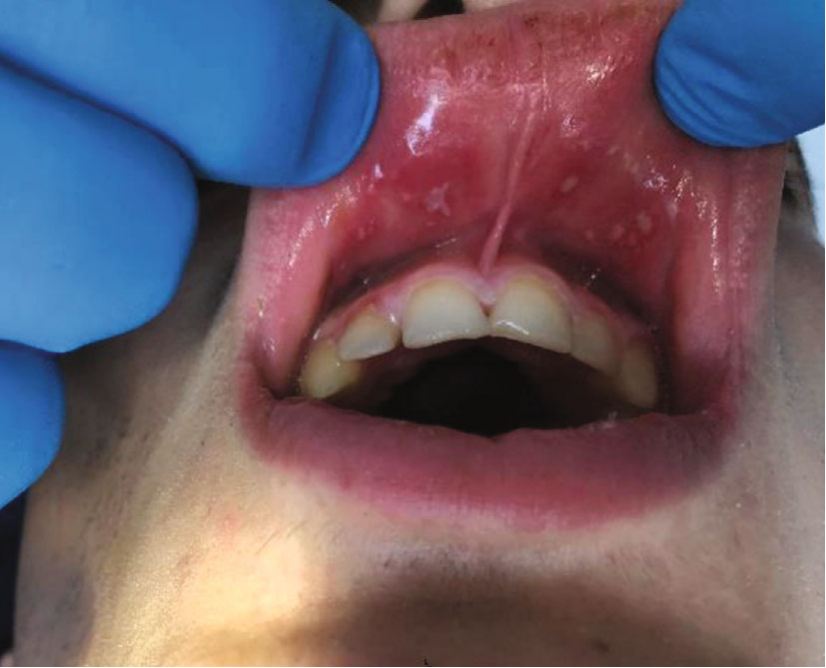
Aphthous ulcers on the mucous membrane of the lips.

On extraoral examination, the upper and lower lips were swollen (Figure 1). Intraoral examination revealed several carious teeth and numerous painful aphthae in the mucous membrane of the lips (Figure 2).

Complete blood count revealed no deviations, and urine test showed leukocytes. Biochemical blood test values were within normal limits, apart from direct bilirubin level -10.6 *μ*mol/L, 8.8 in dynamics, and indirect bilirubin counted 58.6 *μ*mol/L, 40.1 in dynamics.

Abdominal ultrasound revealed that the spleen was 15.8 cm enlarged, the left kidney measured 11.2 cm, and the liver was enlarged and palpable under hypochondrium. There was moderate periportal infiltration and no portal hypertension. The gallbladder and pancreas showed no abnormal changes; all aforementioned organs were of homogenous structure.

Chest radiography did not reveal any abnormalities; echocardiography detected 0-1 mitral valve regurgitation. Antibodies to hepatitis and infections were negative. Specialists in hematology and infectious diseases were consulted and later examined for Gilbert's syndrome (GS) by molecular genetic testing. Homozygous UGT1A1∗28 alleles were identified, confirming the diagnosis of Gilbert's syndrome.

**Figure 3:**
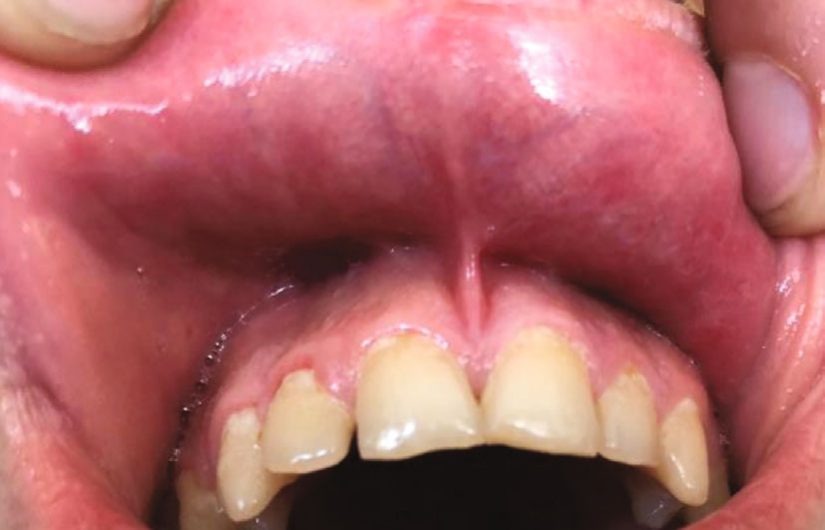
The mucous membrane of the lip with resolution of aphthous ulcers.

Treatment was carried out in collaboration with different medical specialties. Initially, treatment started locally with oral prophylaxis and dental caries management. Aphthous ulcers were treated with DIPLEN-DENTA G and DIPLEN-DENTA X adhesive films. The first one or two applications of adhesive films were performed in the clinic by the treating clinician, and later, it was applied by the patient at home. The patient was trained on how to use these films. The films were applied twice daily until the ulcers healed. In this reported case, aphthous ulcers in the mucous membrane cleared up within two weeks (Figure 3). The patient will be followed up by the general medical practitioner for monitoring any systemic conditions.

## 3. Discussion

Gilbert's syndrome, a benign hereditary hyperbilirubinemia, is caused by reduced UGT1A1 enzyme activity. Excretion of bilirubin is reduced following decreased glucuronidation, a prerequisite for bilirubin elimination. This results in an increase of indirect, or unconjugated, bilirubin (UCB). GS is inherited in an autosomal recessive manner, with a prevalence of 4-16% across varying populations [9]. Patients with GS are usually asymptomatic and require no treatment. Some patients may experience recurrent jaundice; about half of these may suffer from associated symptoms such as abdominal pain, fatigue, and bloating [12]. RAS has, to our knowledge, not been reported in association with GS in the literature.

Recurrent aphthous stomatitis is the most common encountered oral mucosal lesion [13]. Clinical characteristics include oval-shaped ulcers, with red hyperemic margins and a central fibrous coating, in nonkeratinized areas of oral mucosa, frequently affecting the vestibule, lips, and particularly areas subjected to trauma by teeth and solid food [1, 2]. Prodromal signs may include local intraoral burning sensation, fatigue, and mood swings. Relapses are sometimes accompanied by regional lymphadenitis and fever [3, 14]. Symptoms remain local but often change in frequency and severity with age and may include pain during speech and food intake. RAS may recur with certain regularity. Remissions can last for several days, months, or years [2, 3, 14].

The etiology of RAS is still not sufficiently clear [1]. However, several known factors contribute to RAS, including stress, viruses, bacteria, sensitivity to foods and drugs, gastrointestinal disease, vitamin deficiency, impaired immune system, autoimmune diseases, and trauma [1–3]. The commonly discussed etiological factors are genetic, nutritional, and immune-mediated. About 30%-40% of patients give a family history of RAS [2]. An increase in polymorphism of Toll-like receptor 4 has been observed in RAS patients, altering innate immunity and the cellular immune response [15].

Nutritional deficiencies of iron, folic acid, or vitamin B_12_ are implicated in up to 42% of cases [16]. Vitamin B_12_ deficiency has also been reported to cause unconjugated hyperbilirubinemia [17]. Histologically, RAS is characterized by a mucosal ulceration with inflammatory cell infiltrate dominated by T-cells [1]. This suggests that the immune system plays a vital part of RAS formation [2, 3]. RAS has also been found to be seen as oral manifestations of some systemic diseases like Behcet's disease, inflammatory bowel disease like Cohn's disease, and celiac disease [3]. There is no published literature currently which suggests an association between RAS and Gilbert's syndrome.

With regard to our case, the patient sought medical consultation for pain in the hypochondrium and oral cavity. The diagnosis of Gilbert's syndrome was based on the increased levels of direct and indirect bilirubin which was further confirmed by the identification of homozygous UGT1A1∗28 alleles in the individual. Gilbert's syndrome is a diagnosis of exclusion and is established by ruling out the other hepatic causes which result in hyperbilirubinemia [9]. The diagnosis of RAS was based on the clinical presentation of numerous small symmetrical ulcers surrounded by an erythematous halo and the presence of pain. It is possible that the aphthous stomatitis could be a manifestation of Gilbert's syndrome, but there is no evidence to prove this. As this is the first case report to document this concurrence, more such cases need to be documented before we can ascertain any association. Gilbert's syndrome is usually asymptomatic and is aggravated following a triggering factor such as stressful events such as surgery, rigorous exercise or sports activities, fasting, and alcohol intake [10]. There are reports of many cases of Gilbert's syndrome which has been reported during pregnancy [9] and as a postsurgical complication [18]. In our case, the symptoms would have been flared up due to stress considering his age and also the simultaneous occurrence of RAS which has also been associated with stress [3]. The patient experiencing difficulty in eating food could be a factor in further aggravation of Gilbert's syndrome. Hence, proper diet management should be considered while treating the patient. According to a study, the occurrence of jaundice during the aggravation of Gilbert's syndrome affects the quality of life of the individual, and therefore, the management of the patients should include avoidance of such episodes [12]. Knowledge of which medications that can be administered safely in patients with Gilbert's syndrome highlight's the importance of its early diagnosis [18].

Increased levels of Unconjugated Bilirubin (UCB) have been implicated in symptomatic improvements of rheumatoid arthritis, inflammatory bowel disease, and multiple sclerosis [19]. UCB levels are slightly elevated in patients with GS. Anti-inflammatory effects may occur at 2 and 12 mg/dL in adults, while proinflammatory effects, cytotoxicity, and oxidative stress may be evident at high concentrations of around 15–20 mg/dL. UCB is found to be toxic to a number of cell lines, in vitro, including platelets, fibroblasts, and leukocytes [20]. When UCB is unbound to albumin, it can easily enter plasma membranes [21]. This may explain the varying effects of UCB. The immune mediated effects implicated in RAS may therefore be modulated by UCB. Impaired lymphocyte proliferation can be observed at UCB levels as low as 6 mg/dL [19]. The proinflammatory process can additionally cause reduced neutrophil function in terms of impaired phagocytosis, production of oxidative species, and migration [22]. RAS is also associated with defective neutrophil function and reduced number of cells [23].

Research on UCB's impact on oral epithelium is scarce. However, bilirubin is known to accumulate in elastin and can be found in oral mucosa [24]. Considering this occurrence, it can be hypothesized that UCB may cause immune mediated changes inducing aphthous ulcers. The mouth is usually not an area investigated during diagnosis of GS, and RAS associated with GS may therefore go unnoticed. This calls for an interdisciplinary approach involving medical and dental practitioners for the effective diagnosis and management of a patient with Gilbert's syndrome. Addition of more such reports of the concurrent existence of GS and RAS will promote more future studies in this regard and lead to establishment of more diagnostic criteria for GS.

In our patient, DIPLEN-DENTA adhesive films were prescribed. The films consist of two layers. The self-adhesive lower layer, loaded with an active substance (gentamicin sulfate in DIPLEN-DENTA G, chlorhexidine digluconate in DIPLEN-DENTA X), was applied to the affected area. The top layer is a protective coating that protects aphthous ulcers from unfavorable external influence, allowing for less unpleasant oral intake. The films have strong wide spectrum antibacterial effects and are said to be effective [25].

## 4. Conclusion

In this case, we have attempted to present the existence of direct symptoms and clinical findings associated with Gilbert's syndrome and the presence of recurrent aphthous stomatitis in the patient. Since this a novel observation, it too early to directly establish an association between these two disease entities. However, at the same time, the simultaneous presentation of the two should not be taken as chance occurrences. Moving forward, it is recommended that all patients with Gilbert's syndrome should be thoroughly evaluated for any oral manifestations in order to effectively manage the patient. Early management of recurrent aphthous stomatitis may also be beneficial in reducing the trigger factors associated with Gilbert's disease.

## Data Availability

All the relevant data pertaining to the diagnosis of this case has been disclosed in this case presentation.
